# Supplementary material for: Competing basal ganglia pathways determine the difference between stopping and deciding not to go
Source: eLife. 2015 Sep 24;4:e08723. doi: 10.7554/eLife.08723 (PMC4686424; doi:10.7554/eLife.08723)
Supplement: Supplementary file 1. — Coordinates are centers of mass for the cluster in MNI-space. N is the number of voxels in each cluster. Values in the left six columns show average condition-wise (general linear model) GLM coefficients and standard deviation across subjects is in parentheses. DOI: http://dx.doi.org/10.7554/eLife.08723.017 [file elife-08723-supp1.docx]

| **Hemisphere** | **Region** | **x** | **y** | **z** | **N** | **No-Go**  **0** | **No-Go 25** | **No-Go 50+** | **Go**  **50-** | **Go**  **75** | **Go**  **100** |
| --- | --- | --- | --- | --- | --- | --- | --- | --- | --- | --- | --- |
| Right | Inferior Frontal Gyrus | 43 | 20 | 12 | 2830 | 1.40 (1.25) | 2.63 (1.37) | 3.32  (1.84) | 3.56 (1.50) | 3.05 (1.26) | 2.01 (1.35) |
| Right | Middle Frontal Gyrus | 38 | 48 | -10 | 83 | -0.23  (1.24) | 0.23 (1.40) | 0.64  (1.27) | 1.24 (1.79) | 0.88 (1.80) | 0.10 (1.72) |
| Right | Thalamus | 9 | -16 | 3 | 154 | 1.78 (1.43) | 2.46 (1.62) | 2.93  (2.29) | 3.87 (2.01) | 3.90 (2.09) | 2.80 (1.64) |
| Right | Caudate | 13 | 10 | 6 | 55 | 0.21 (1.44) | 1.60 (1.88) | 1.66  (2.22) | 2.36 (2.38) | 2.30 (1.55) | 1.94 (1.51) |
| Right | Putamen | 31 | -11 | 4 | 44 | 0.11 (0.85) | -0.50 (1.30) | -0.64 (1.33) | 0.09  (1.53) | 0.45  (1.05) | 0.75 (1.45) |
| Right | Middle Temporal Gyrus | 49 | -66 | 26 | 60 | -1.33 (2.14) | -2.00 (2.56) | -2.93 (3.39) | -2.34 (2.36) | -1.59 (2.93) | -0.47  (2.74) |
| Right | Inferior Parietal Lobule | 48 | -44 | 43 | 1400 | 1.23 (1.28) | 2.27 (1.22) | 3.26  (1.46) | 3.26 (1.87) | 3.06 (1.35) | 2.06 (1.77) |
| Right | Superior Frontal Gyrus | 21 | 49 | 31 | 45 | -0.67 (1.23) | 0.40 (1.37) | 0.40  (1.73) | 0.90 (1.15) | 0.50 (1.58) | 0.26 (1.00) |
| Right | Precuneus | 12 | -67 | 42 | 83 | 2.44 (2.78) | 3.84 (2.77) | 3.70  (3.12) | 5.09 (4.42) | 4.64 (3.90) | 3.47 (3.24) |
| Left | Inferior Temporal Gyrus | -56 | -10 | -20 | 44 | -1.31 (1.69) | -1.61 (1.82) | -2.82 (3.60) | -2.84 (2.53) | -1.89 (1.87) | -1.00 (1.77) |
| Left | Fusiform Gyrus | -43 | -60 | -17 | 84 | 3.16 (2.00) | 3.80 (1.51) | 4.63  (2.07) | 3.80 (2.38) | 3.69 (1.61) | 3.40 (1.33) |
| Left | Inferior Frontal Gyrus | -37 | 18 | -4 | 912 | 1.02 (1.32) | 2.11 (1.15) | 3.03  (1.98) | 3.60 (1.39) | 2.68 (1.01) | 1.70 (1.20) |
| Left | Medial Frontal Gyrus | -3 | 50 | -9 | 477 | -1.59 (1.88) | -2.35 (2.24) | -3.76 (3.55) | -3.44 (2.22) | -2.31 (1.91) | -1.59 (2.10) |
| Left | Ventral Striatum | -1 | 16 | -9 | 100 | -0.32 (1.03) | -0.95 (1.70) | -1.84 (2.35) | -1.45 (1.23) | -0.73 (0.97) | -0.09 (1.07) |
| Left | Thalamus | -6 | -16 | -2 | 72 | 1.02 (1.00) | 1.56 (1.18) | 2.36  (2.18) | 2.73 (1.08) | 2.68 (1.00) | 2.15 (1.03) |
| Left | Putamen | -27 | -13 | 7 | 48 | 0.13 (1.00) | -0.22 (1.66) | -0.75 (1.86) | 0.21  (1.83) | 0.97  (1.56) | 1.48 (1.88) |
| Left | Insula | -39 | -3 | 7 | 41 | 0.63 (1.20) | 1.25 (1.21) | 2.29  (1.84) | 3.08 (1.68) | 3.09  (1.33) | 2.94 (1.32) |
| Left | Inferior Frontal Gyrus | -44 | 9 | 29 | 426 | 1.40 (1.50) | 2.03 (1.55) | 3.22  (1.96) | 2.77 (2.22) | 2.36 (1.59) | 1.32 (1.94) |
| Left | Angular Gyrus | -44 | -72 | 30 | 328 | -1.87 (1.80) | -2.77 (2.17) | -3.40 (2.83) | -3.77 (2.25) | -2.49 (1.63) | -1.71 (1.89) |
| Left | Superior Frontal Gyrus | -9 | 57 | 35 | 128 | -1.51 (1.95) | -2.88 (2.81) | -3.27 (3.50) | -3.67 (2.29) | -2.79 (1.56) | -1.90 (2.59) |
| Left | Inferior Parietal Lobule | -34 | -52 | 46 | 459 | 1.64 (1.60) | 2.11 (1.30) | 2.81  (1.33) | 2.99 (2.04) | 2.73 (1.67) | 1.76 (1.76) |
| Bilateral | Posterior Cingulate | -2 | -56 | 22 | 957 | -0.68 (1.97) | -1.84 (2.43) | -2.70 (3.29) | -2.47 (2.97) | -0.82 (2.20) | -0.23 (2.14) |
| Bilateral | White Matter (Callosum) | 3 | -23 | 29 | 208 | 1.97 (1.46) | 3.28 (1.69) | 2.99  (1.81) | 3.85 (2.34) | 3.58 (1.68) | 2.57 (1.33) |
| Bilateral | preSMA | 4 | 21 | 47 | 1952 | 1.19 (1.32) | 2.31 (1.07) | 2.92  (2.03) | 3.39 (1.24) | 2.60 (1.04) | 1.91 (.94) |
